# Studies on the regulatory mechanism of isocitrate dehydrogenase 2 using acetylation mimics

**DOI:** 10.1038/s41598-017-10337-7

**Published:** 2017-08-29

**Authors:** Yuqun Xu, Lingwen Liu, Akira Nakamura, Shinichi Someya, Takuya Miyakawa, Masaru Tanokura

**Affiliations:** 1 Laboratory of Basic Science on Healthy Longevity, Department of Applied Biological Chemistry, Graduate School of Agricultural and Life Sciences, The University of Tokyo, 1-1-1 Yayoi, Bunkyo-ku, Tokyo, 113-8657 Japan; 2 Departments of Aging and Geriatric Research, University of Florida, Gainesville, Florida 32610-0143 United States of America

## Abstract

Mitochondrial isocitrate dehydrogenase 2 (IDH2) converts NADP^+^ to NADPH and promotes regeneration of reduced glutathione (GSH) by supplying NADPH to glutathione reductase or thioredoxin reductase. We have previously shown that under calorie restriction, mitochondrial deacetylase Sirt3 deacetylates and activates IDH2, thereby regulating the mitochondrial glutathione antioxidant defense system in mice. To investigate the regulatory mechanism of mIDH2 (mouse mitochondrial IDH2), we used lysine-to-glutamine (KQ) mutants to mimic acetylated lysines and screened 15 KQ mutants. Among these mutants, the activities of the K256Q and K413Q proteins were less than 50% of the wild-type value. We then solved the crystal structures of the wild-type mIDH2 and the K256Q mutant proteins, revealing conformational changes in the substrate-binding pocket. Structural data suggested that positively charged Lys256 was important in stabilizing the pocket because it repelled a lysine cluster on the other side. Glutamine (or acetylated lysine) was neutral and thus caused the pocket size to decrease, which might be the main reason for the lower activity of the K256Q mutant. Together, our data provide the first structure of an acetylation mimic of mIDH2 and new insights into the regulatory mechanism of acetylation of mIDH2.

## Introduction

The isocitrate dehydrogenases (IDHs) compose a family of enzymes that catalyze the sequential dehydrogenation and decarboxylation of isocitrate to α-ketoglutarate (αKG) (Supplementary Fig. 1a) in the mitochondria or the cytosol. Isocitrate dehydrogenase 2 (IDH2) is a mitochondrial NADP^+^-dependent IDH that converts NADP^+^ to NADPH and promotes regeneration of reduced glutathione (GSH) or reduced thioredoxin by supplying NADPH to glutathione reductase or thioredoxin reductase (PMID: 11278619; 21094524). Hence, IDH2 plays an important role in reactive oxygen species (ROS) removal in the mitochondria or the prevention of age-related diseases^1–3^. IDH2 is also critical in cell proliferation. Mutations at Arg140 and Arg172 of IDH2 are accompanied by 2-hydroxyglutarate (2HG) accumulation that is associated with cancer^4, 5^.

We have previously shown that under calorie restriction (CR), mitochondrial deacetylase Sirt3 deacetylates and activates IDH2 in mouse cochlea^3^. A study applying a quantitative acetyl-proteomic method^6^ provided the evidence that Sirt3 played an important role in regulating metabolic reprogramming in response to CR by deacetylating proteins, including IDH2. Regulation of the acetylation status of multiple proteins by Sirt3 led to more efficient fuel utilization and energy production, which might be related to life span extension. We have reported that enhanced activity of IDH2 increased NADPH levels in the mitochondria, which in turn led to the activation of NADPH-dependent glutathione reductase. This change was associated with decreased levels of ROS and prevention of age-related hearing loss (AHL) in mammals^3, 7–11^ (Supplementary Fig. 1b). Reducing food consumption without causing malnutrition consistently extends life spans and retards age-associated diseases in a variety of species, including yeast, fish, hamsters, dogs, mice and monkeys, as demonstrated by studies since the early 20th century^12–22^. Therefore, acetylation/deacetylation of IDH2 may play a key role in the regulation of cell survival and life span.

Several possible lysine acetylation sites in IDH2 have been reported in several proteomic studies, and a wide range of acetylation sites are purported to be involved^9, 23–28^. For example, a previous proteomic survey identified Lys106 of mIDH2 (mouse mitochondrial IDH2) as a lysine acetylation site in both fasted and fed samples and Lys272 in the fasted sample only^23^. Another study, using rigorous label-free quantitative MS, analyzed changes in lysine acetylation from mouse liver mitochondria and identified 21 lysine acetylation sites in mIDH2. Among them, only Lys360 showed a significant increase in acetylation in Sirt3^−/−^ mice^24^. In another study^6^, a quantitative mass spectrometry method was used to examine the global mitochondrial protein acetylome during CR and/or knockout of Sirt3, and acetylation of K413 in mIDH2 increased 50% in Sirt3^−/−^, proving that K413 was a target site of Sirt3. However, it is not clear which lysine acetylation sites play a more critical role in modulating mIDH2 activity and how mIDH2 activity is regulated by acetylation and deacetylation.

To better understand which lysine acetylation sites regulate mIDH2 activity, we created a series of lysine-to-glutamine (KQ) mutations to mimic the acetylated states in mIDH2 *in vitro*. This method is effectively used in many studies to mimic the acetylation of lysine^25, 29, 30^ (Supplementary Fig. 1c). We measured the activity of these acetylation mimics to probe for regulatory acetylation sites. Importantly, we solved the crystal structure of an acetylation mimic of mIDH2 (K256Q mutant). Structural changes have been observed in a cleft between the large and small domains, and we subsequently found that the K256Q mutant has a smaller substrate-binding pocket. These results show the structural and functional impact of lysine acetylation on mIDH2 and enable the further study of acetylation/deacetylation regulation of mIDH2.

## Results

### Screening of lysine acetylation sites related to the regulation of mIDH2 activity

**Figure 1:**
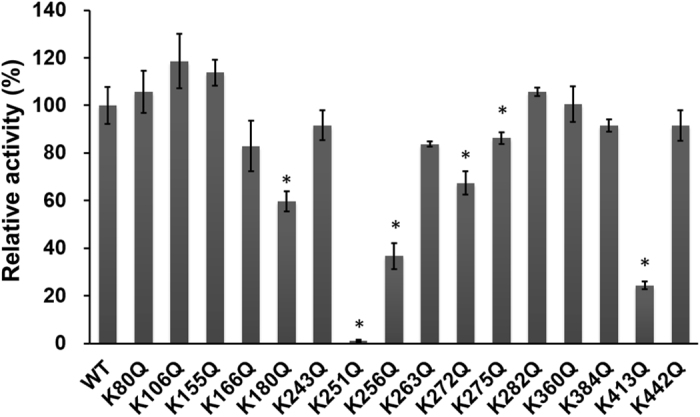
Screening of regulatory lysine in mIDH2 using acetylation mimics. The activity of the KQ mutants compared to that of wild-type mIDH2 is shown. Measuring the activity of each mutant was repeated three times, which served as three technical replicates. The final enzyme concentration was 5 nM, and isocitrate and NADP^+^ were added at their saturating concentrations of 1 mM. The data represent the means ± SD, and Student’s *t*-test was used to determine whether differences in values relative to those of wild-type mIDH2 were significant; significance is denoted by an asterisk (*p* < 0.05).

To discriminate the regulatory acetylation site(s) in mIDH2, 15 acetylation mimics (K80Q, K106Q, K155Q, K166Q, K180Q, K243Q, K256Q, K263Q, K272Q, K275Q, K282Q, K360Q, K384Q, K413Q, and K442Q mutants) were generated by site-directed mutagenesis. In addition, K251Q was used as a negative control because Lys251 has been reported to be a critical residue in the catalytic activity of porcine IDH2^31^. The relative activity of mutants, compared to that of the wild-type mIDH2, was measured by monitoring the absorption of NADPH product at 340 nm. A significant decrease in activity was observed in the K180Q, K251Q, K256Q, K263Q, K272Q, K275Q and K413Q mutants (Fig. 1). The K251Q, K256Q and K413Q mutants exhibited an ~50% decrease in IDH2 activity compared to that of wild type. Consistent with a previous report^25^, the K413Q mutant exhibited a significant loss in activity, but the K256Q mutant showed less activity loss, probably due to the use of a different expression system and the mIDH2 proteins being of different purity. The lowest activity level was observed in the K251Q mutant, suggesting that Lys251 also plays a crucial role in the catalytic activity of mIDH2. Although Lys360 and Lys384 were reported to be probable Sirt3-dependent deacetylation sites in murine Sirt3 knockout cells^24, 26^, their acetylation mimics did not exhibit significant decreases in IDH2 activity.

### Contribution of Lys256 and Lys413 to the mIDH2 reaction

To determine the *K*_m_ and *k*_cat_ values of isocitrate and NADP^+^ for the two most affected mutants, K256Q and K413Q, we conducted a steady-state kinetics analysis. The *k*_cat_ value of the K256Q mutant decreased two-fold, but the mutant’s *k*_cat_/*K*_m_ value (catalytic efficiency) for isocitrate was not significantly different from that of wild-type mIDH2 (Supplementary Fig. 2 and Table 1). Similarly, at saturating concentrations of the substrate isocitrate, the *V*_max_ value of the K256Q mutant was two-fold lower than that of the wild type, as was its *K*_m_ value; in addition, the *k*_cat_/*K*_m_ value remained unchanged for the cofactor NADP^+^. Together, these results suggest a link between the decreased *K*_m_ and *k*_cat_ values of the K256Q mutant and a reduction in the final step of catalysis or the enzyme-substrate complex breaking down to form product and free enzyme^32^. This conclusion could also be derived from a lower catalytic reaction rate and/or slower release of product. Therefore, Lys256 may affect the catalytic reaction of mIDH2.

**Table 1:** Kinetic parameters for the activity of wild-type mIDH2 (WT) and the K256Q and K413Q mutants.

Enzyme	*K*_m_ (μM)	*V*_max_ (μM s^−1^)	*k*_cat_ (s^−1^)	*k*_cat_ */K*_m_ (s^−1^ μM^−1^)
(Kinetic parameters for isocitrate)				
WT	54.4 ± 5.6^a^	0.028 ± 0.001	5.60 ± 0.14	0.111 ± 0.011
K256Q	30.4 ± 2.3	0.013 ± 0.0002	2.66 ± 0.04	0.088 ± 0.008
K413Q	55.5 ± 7.1	0.023 ± 0.001	4.61 ± 0.15	0.083 ± 0.008
(Kinetic parameters for NADP^+^)				
WT	33.7 ± 2.4	0.031 ± 0.0006	6.36 ± 0.14	0.186 ± 0.011
K256Q	14.8 ± 1.3	0.015 ± 0.0003	3.13 ± 0.13	0.205 ± 0.022
K413Q	112.2 ± 13.2	0.033 ± 0.001	7.69 ± 0.49	0.059 ± 0.001

^a^Values are the means of four technically independent replicates and are displayed as the means ± SD.

At saturating concentrations of NADP^+^, the K413Q mutant showed no significant changes in its *K*_m_ and *k*_cat_ values for isocitrate from those of the wild-type mIDH2, which is consistent with a previous report^25^. In contrast, the *k*_cat_/*K*_m_ value for NADP^+^ of the K413Q mutant was only 30% of the corresponding value of wild-type mIDH2; this decrease arose from a 3-fold increase in the *K*_m_ value. This observation is consistent with a previously reported^25^ 5-fold increase in the *K*_m_ value. Together, our data and previous reports suggest that Lys413 is important in NADP^+^ binding and/or electron transfer.

### Overview of the structure of mIDH2

To investigate the functional effect of lysine acetylation on mIDH2, we determined the crystal structures of wild-type and K256Q mIDH2 (Table S1). Although the K413Q mutation appears to have a more striking effect on activity, we were not able to solve the crystal structure of this mutant. The crystal structure of wild-type mIDH2 was determined at 3.3 Å resolution by using a molecular replacement method using porcine IDH2 (PDB code: 1LWD) as a starting model and was found to belong to space group *P*3_2_21. On the other hand, the structure of the K256Q mutant was solved at 2.3 Å resolution and belonged to space group *P*3_2_. The models were refined to *R*_work_ and *R*_free_ values of 19.2 and 22.2% (wild type) and 20.9 and 24.3% (K256Q), respectively.

**Figure 2:**
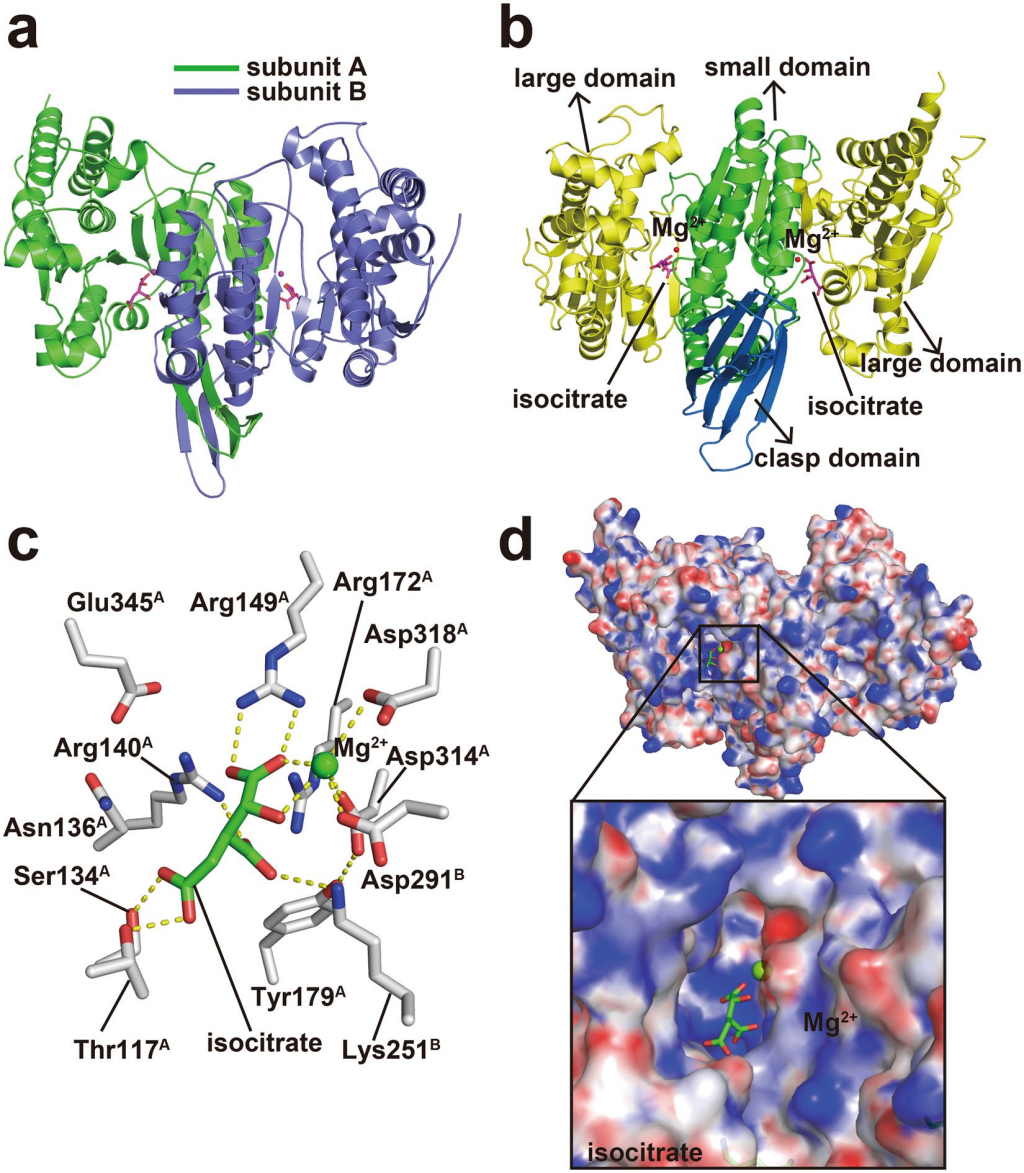
Substrate-binding pocket of mIDH2. (**a**) Structural overview of wild-type mIDH2. Wild-type mIDH2 is a homodimer with two subunits, one colored green and one slate blue. An isocitrate molecule and a Mg^2+^ ion were bound in each subunit and are represented by the magenta stick and magenta sphere, respectively. (**b**) Conserved domains of mIDH2. The large domain (residues 40–152 and 327–452), the small domain (residues 153–180 and 224–326) and the clasp domain (residues 181–223) are shown in yellow, green and blue, respectively. (**c**) Residues involved in isocitrate and Mg^2+^ binding (subunit A). Isocitrate is shown as a green stick, and Mg^2+^ is shown as a green sphere. Amino acid residues are represented by white sticks. Blue and red indicate nitrogen and oxygen atoms, respectively. Superscript capital letters for amino residues represent which subunit they are from. Hydrogen bonds are represented using dashed lines. (**d**) The electrostatic surface of wild-type mIDH2. The surface view of the overall structure (left panel) and a close-up view of the substrate-binding pocket (right panel) are shown. The electrostatic potential of the surface is colored in a range from −12 kTe^−1^ (red) to + 12 kTe^−1^ (blue). Isocitrate and Mg^2+^ are shown in green.

In the crystal structure of wild-type mIDH2, two molecules were found to form a dimer in the asymmetric unit (Fig. 2a), which was also found to occur in previously reported structures of human IDH2 and porcine IDH2. The two subunits share highly similar structures (root-mean-square deviation (RMSD) of 0.3 Å for the superposition of 826 C_α_ atoms) (Supplementary Fig. 3a). In each subunit, one isocitrate molecule and Mg^2+^ were similarly bound in the active site (isocitrate-binding pocket). Similar to other isocitrate dehydrogenases^33–36^, mIDH2 consists of three domains: a large domain, a small domain and a clasp domain (Fig. 2b). A hydrophilic cleft is formed between the large domain and the small domain and serves as the active site where the isocitrate molecule and Mg^2+^ are bound. Amino acid residues, including Thr117, Ser134, Arg140, Arg149, Lys251, Asp291, Asp314 and Asp318, was involved in isocitrate and Mg^2+^ binding by hydrogen bonding (Fig. 2c and Supplementary Fig. 3b). It is noteworthy that two Arg residues, Arg140 and Arg172, are thought to be crucial for IDH2 activity because their frequent mutations have been observed in several cancer-associated diseases^4, 5^. Arg residues (Arg140, Arg149 and Arg172) and Lys251 on one side stabilized the binding of negatively charged isocitrate, while Asp residues (Asp291, Asp314 and Asp318) on the other side stabilized the positively charged Mg^2+^ metal ion by electrostatic interactions (Fig. 2d). As expected, mutation of Lys251 to Gln led to complete loss of IDH2 activity, demonstrating the importance of these active site residues.

The structure of mIDH2 resembles the closed form of isocitrate-bound porcine IDH2 (PDB code: 1LWD)^33^ and exhibits an RMSD value of 1.3 Å in structure alignments. A structural segment, namely, the putative phosphorylation loop (residues 115–134) at the isocitrate-binding pocket, was suggested to form a loop conformation in the open, inactive form and an α-helix in the closed, active form^33, 37^ (Supplementary Fig. 4). This segment of mIDH2, when complexed with isocitrate, formed a helix and therefore adopted a closed conformation.

### Structural changes observed in the K256Q mutant

**Figure 3:**
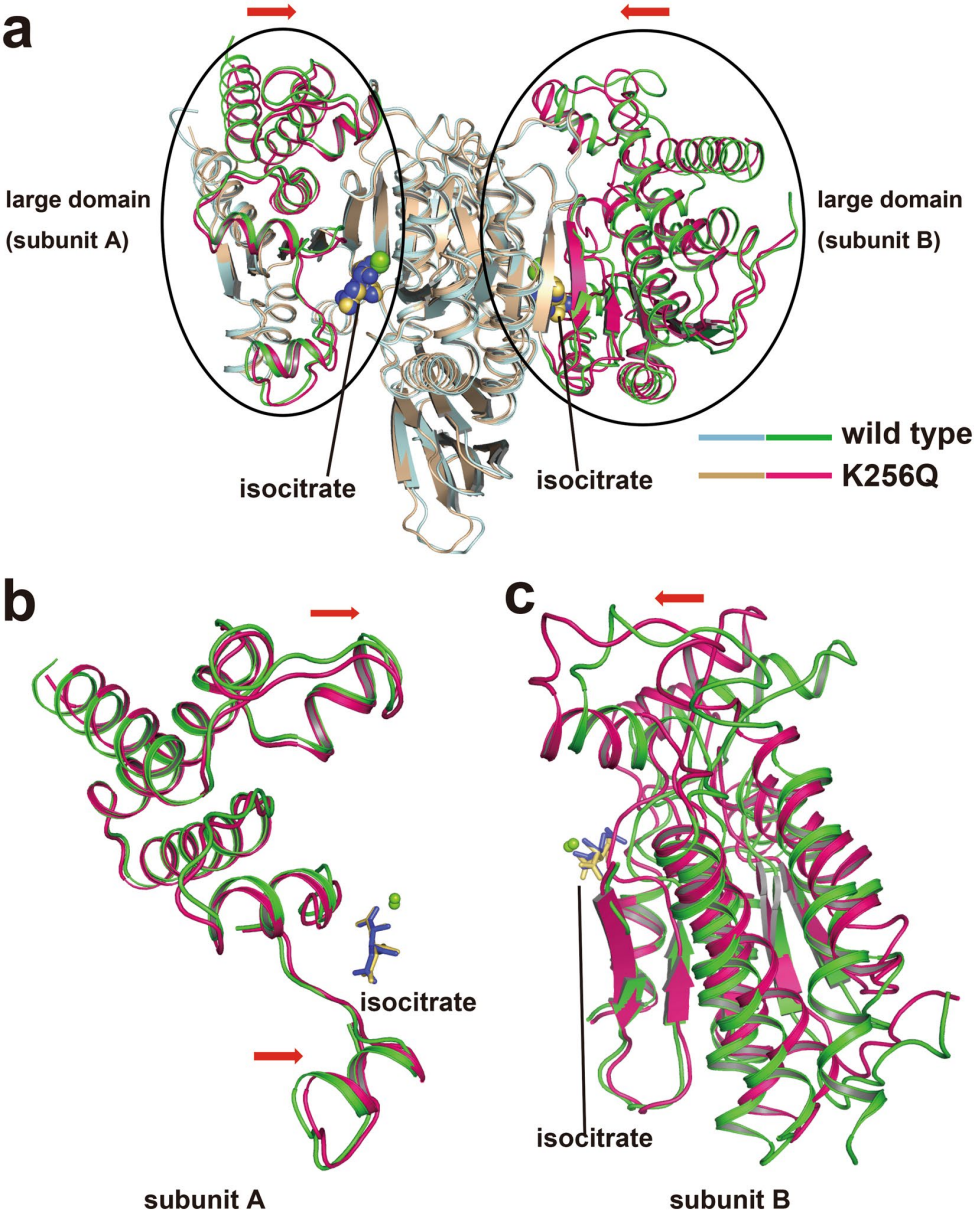
Structural comparison of wild-type mIDH2 and the K256Q mutant. (**a**) Structural alignment of the wild-type mIDH2 (light blue) and the K256Q mutant (tan) proteins. The two circles show conformational differences between the two structures. The regions that are structurally different in the large domain have been highlighted with different colors (green for wild-type mIDH2 and magenta for the K256Q mutant). (**b,c**) Enlarged view of large domains from the structural overlay of wild-type mIDH2 and the K256Q mutant for subunits A (**b**) and B (**c**), respectively. Red arrows represent the structural differences in K256Q compared to the wild-type structure. Mg^2+^ ions are represented by green spheres, and isocitrate molecules are represented by purple sticks and yellow sticks in wild-type mIDH2 and the K256Q mutant, respectively.

**Figure 4:**
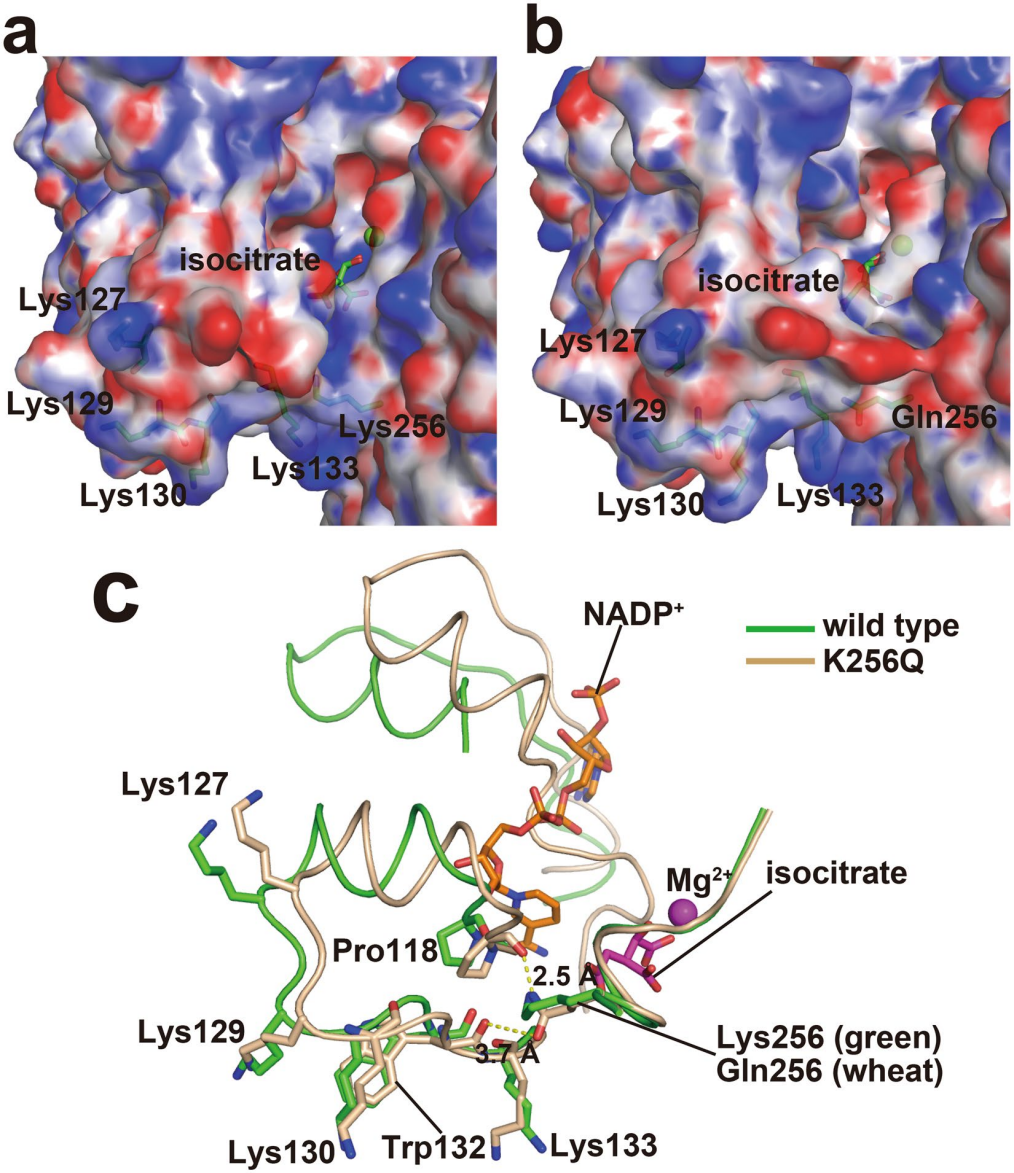
Detailed structural differences between wild-type mIDH2 and the K256Q mutant. (**a**,**b**) A comparison of the electrostatic surfaces of wild-type mIDH2 (**a**) and the K256Q mutant (**b**) in the vicinity of the substrate-binding pocket. Isocitrate and Mg^2+^ ions are shown in green. The lysine cluster (Lys127, Lys129, Lys130 and Lys133), Lys256 and Gln256 are indicated by green sticks. (**c**) The lysine cluster near Lys256 and Gln256. After structural alignment of wild-type mIDH2 (green) and the K256Q mutant (tan), structural differences near the substrate-binding pocket are obvious. Isocitrate and Mg^2+^ ions are colored in magenta. The structures of mIDH2 are superimposed with the crystal structure of human IDH2 (PDB code: 4JAS) and NADP^+^ (from the structure of human IDH2) is shown in orange. Hydrogen bonds are indicated by yellow dashed lines, and bond lengths are shown in black (Å).

To investigate the molecular mechanism of Lys256-regulation of mIDH2 activity, the structure of the K256Q mutant was compared to that of wild-type mIDH2. No large differences existed in the small domains and clasp domains (RMSD of 0.9 Å for a superposition of 818 C_α_ atoms). The isocitrate molecule, Mg^2+^ ion, and the binding residues overlapped well in the wild-type enzyme and the K256Q mutant (Supplementary Fig. 5). However, the regions involving N-terminal residues 40‒145 and C-terminal residues 329‒451 in the large domain of the K256Q mutant were closer to the isocitrate-binding pocket (Fig. 3). In the structure of wild-type mIDH2, Lys256 did not bind isocitrate and Mg^2+^ directly but was adjacent to the isocitrate-binding pocket (Fig. 3b and c), which might importantly stabilize the pocket conformation by regulating the arrangement of the large domain. Compared to wild-type mIDH2, the K256Q mutant had a smaller binding pocket, which was observed in the surface view of the structures (Fig. 3b and c). Using the CASTp server^38^, we estimated the volume of the isocitrate-binding pocket. In contrast to the wild-type subunit A and B volumes of 3304 and 4244 Å^3^, respectively, the K256Q mutant subunit A and B volumes were 2895 and 3135 Å^3^, respectively. These results show an apparent reduction in the pocket size of the K256Q mutant. The surface electrostatic potential of both structures showed that substitution of Lys256 to Gln led to the presence of fewer positive charges (Fig. 4a and b). We note that because there are four positively charged lysine residues in the large domain, consisting of Lys127, Lys129, Lys130 and Lys133, those positively charged lysine residues might exert repulsion on Lys256 in the small domain, resulting in a wider cleft. Concordantly, the lysine-to-glutamine (KQ) substitution, mimicking acetylated states, could neutralize the positive charge, resulting in a narrower cleft and a smaller isocitrate-binding pocket. Furthermore, the shortened distance enabled Gln256 to form hydrogen bonds with the carbonyl groups of Pro118 (2.5 Å) and Trp132 (3.7 Å), which eventually enhanced the conformational change (Fig. 4c). In contrast, the corresponding distances in wild-type mIDH2 are both 4.2 Å, which are too distant to form hydrogen bonds. Therefore, these results suggest that a stronger negative charge distribution near Gln256 and the smaller substrate pocket might have increased the difficulty of releasing product from the pocket, resulting in slower turnover (lower *k*_cat_).

## Discussion

Mutations in IDH2 have been shown to be tumorigenic, resulting in glioma, chondrosarcoma and acute myeloid leukemia^4, 5^. *IDH2* deficiency also results in increased insulin sensitivity, resistance to obesity^39^, and impaired vascular function^40^. Under calorie restriction conditions, IDH2 regulates the mitochondrial glutathione antioxidant defense by supplying NADPH to glutathione reductase in mouse cochlea^41^ (Supplementary Fig. 1b). In this CR-mediated pathway, we have shown that mIDH2 activity was regulated by its acetylation/deacetylation state. To date, a wide range of the acetylated sites of IDH2 have been identified by numerous proteomic studies^12–22^. In the current study, lysine-to-glutamine (KQ) substitutions were used to mimic the acetylated states of mIDH2 *in vitro*, and the resulting activity was measured to determine the molecular mechanism of the regulatory lysine site(s). The K251Q, K256Q and K413Q mutants were observed to lose more than 50% of wild-type activity. Of these mutants, the K251Q mutant showed a complete loss of catalytic activity, suggesting that Lys251 might be the catalytic residue in mIDH2. In agreement, an alignment of amino acid sequences and crystal structures of mIDH2 and porcine IDH2 indicated that Lys251 of mIDH2 corresponded to Lys212 of porcine IDH2, which has been reported to be the critical residue in its catalytic activity^31^. Lys413 was located near the NADP^+^-binding pocket, which may affect NADP^+^ binding when it is mutated to a Gln residue (Supplementary Fig. 6). In agreement with this observation, the K413Q mutant showed a much lower *k*_cat_/*K*_m_ value for NADP^+^ than the wild-type mIDH2 did. Furthermore, the K180Q, K272Q and K275Q mutants also exhibited reduced activity, albeit to a lesser extent. These residues were located at the small domain near the isocitrate-binding pocket (Supplementary Fig. 7), indicating that they are likely involved in the catalytic reaction of mIDH2. The surface-located residue Lys360 has been reported to show a significant increase in the frequency of acetylation changes in the absence of Sirt3^24^. However, concordant with the structural analysis, our activity measurement showed that the activity of the acetylation mimic K360Q was identical to that of wild type.

To investigate the mechanism that determines how the acetylation of Lys256 regulates the activity of mIDH2, we compared the crystal structures of wild-type mIDH2 and the K256Q mutant, both complexed with isocitrate and Mg^2+^. Structural differences were observed in a hydrophilic cleft that was formed between the large and small domains. This cleft is the active site where the isocitrate molecule and Mg^2+^ ion bind. In the crystal structure of the K256Q mutant, a narrower cleft and smaller pocket were observed. Lys256 was located in the small domain and was in the vicinity of the isocitrate-binding site, which is important to stabilize the pocket conformation. We also found that positively charged Lys256 repulsed the lysine cluster (Lys127, Lys129, Lys130 and Lys133) in the large domain. We speculate that when Lys256 was converted to Gln256, charge neutralization could have weakened the repulsion, resulting in a narrower cleft and smaller pocket. As a result, Gln256, in the small domain, was able to form hydrogen bonds with Pro118 in the large domain, which maintained the shortened distance between the two domains. Moreover, acetylation of a lysine residue also resulted in this charge neutralization. Our crystal structure could therefore mimic the structural changes resulting from the acetylation of Lys256. In addition, desuccinylation of mIDH2 by another member of the sirtuin family, Sirt5, also promoted ROS removal and maintain NADPH homeostasis^42^. In total, 15 succinylation sites on mIDH2, including K256, have been reported^43^. Succinylated K256 possessed a negative charge, which might also bring about similar conformational changes to regulate the activity of IDH2. All of these succinylation sites overlap with acetylation sites, which frequently occurs among enzymes involved in the TCA cycle, the urea cycle and fatty acid oxidation^44^. Crosstalk between post-translational modifications has been reported to occur in histones, signaling proteins and metabolic enzymes^45–47^. In the case of mIDH2, deacetylation by Sirt3 and desuccinylation by Sirt5 can both activate mIDH2. This dual regulation by distinct sirtuins suggests a crosstalk or synergy between different post-translational modifications. Although the acetylation levels and succinylation levels could be dynamically affected by growth conditions^44, 48^, the two converged regulation pathways might synergistically regulate activity of mIDH2.

The structural difference between wild-type mIDH2 and the K256Q mutant is more obvious in subunit B than in subunit A, although this difference might be caused by crystal packing (Fig. 4a and Supplementary Fig. 8). Moreover, our study of the structure and function of acetylation mimic K256Q revealed the molecular mechanism of inactivation by acetylation and partially elucidated the activity regulation of lysine acetylation/deacetylation in mIDH2. Further comprehensive mapping of lysine acetylation/deacetylation in mIDH2 and further investigation of the molecular mechanism of deacetylation by Sirt3 are of great importance and interest.

## Methods

### Cloning, overexpression and purification of recombinant proteins

A DNA fragment of mouse IDH2 (mIDH2, UniProtKB accession No. P54071) without 39 N-terminal amino acids that were truncated was amplified from mouse liver total RNA using PrimeScript II High Fidelity RT-PCR kit (Takara). The amplified DNA fragment was cloned into the EcoRI/HindIII site of the pET-26b ( + ) plasmid encoding a C-terminal histidine tag (Novagen). The QuickChange II site-directed mutagenesis kit (Agilent) was used to generate acetylation mimic mutants (K80Q, K106Q, K155Q, K166Q, K180Q, K243Q, K251Q, K256Q, K263Q, K272Q, K275Q, K282Q, K360Q, K384Q, K413Q, and K442Q). K251Q was used as a negative control because of its critical role in porcine IDH2 activity^31^. Other selected lysine sites have been reported to be possible acetylation sites by several additional studies^9, 23–28^. The wild-type and mutant mIDH2 proteins were overexpressed in *E. coli* KRX (Promega).

For purification, harvested cells were lysed by sonication in a solution containing 50 mM Tris-HCl (pH 7.4), 10% glycerol, 200 mM NaCl, 4 mM MgCl_2_ and 10 mM imidazole. After the lysed cells were centrifuged, the supernatant was collected and applied to a Ni-NTA resin (Qiagen). After washing the resin with buffer containing 50 mM imidazole, protein was eluted with a buffer containing 200 mM imidazole. The eluted mIDH2 protein solution was further purified using a Resource S column (GE Healthcare) that had been preequilibrated with a solution containing 20 mM MES-HCl (pH 6.5), 5% glycerol and 4 mM MgCl_2_ and was eluted with a linear gradient of 0−1 M NaCl. For crystallization, a buffer containing 20 mM Tris-HCl (pH 7.4), 200 mM NaCl, 4 mM MgCl_2_, 1 mM DTT and 10 mM isocitrate was used.

### Measurement of isocitrate dehydrogenase activity

The activity of mIDH2 was measured by using a Tecan Infinite M1000 monochromator to monitor the reduction of NADP^+^ to NADPH, which was calculated from its UV absorbance at 340 nm. To map the activity of the lysine-to-glutamine (KQ) substitution mutants, we prepared reaction mixtures by adding 10 μl of 20 mM NADP^+^, 10 μl of 20 mM isocitrate and 170 μl of assay buffer (20 mM Tris-HCl, pH 7.4 and 1 mM MgCl_2_) to 10 μl of 0.1 μM mIDH2 enzyme in flat-bottomed, transparent 96-well plates. The absorbance at 340 nm was read every 30 seconds for 60 min at room temperature. The concentration of the NADPH product was calculated by constructing a standard curve of NADPH. The initial velocity (*v*_0_) was determined from the slope of the graphs of concentration versus time during the first 5 min. Relative activity was estimated by dividing the *v*_0_ of each KQ mutant by that of wild type.

To determine the *K*_m_ and *k*_cat_ values of mIDH2 catalyzing isocitrate decarboxylation, the concentrations of isocitrate were 1.00, 0.460, 0.230, 0.116, 0.058 and 0.029 mM, while the NADP^+^ concentration was maintained at the saturating level of 1.00 mM. To determine the *K*_m_ and *k*_cat_ values of NADP^+^ reduction, the concentrations of NADP^+^ were 1.000, 0.243, 0.081, 0.027, 0.009 and 0.006 mM, while isocitrate was added to the saturating concentration of 1 mM. Reaction progress was monitored by measuring the absorbance at 340 nm every 30 seconds for 60 min at room temperature. The progress curves of reactions are shown in Supplementary Fig. 9. *v*_0_ was determined from the slope of the graphs of concentration versus time during the first 5 min. The *K*_m_ values and their standard deviations (SD) were determined from Michaelis-Menten plots.

### Crystallization and data collection

Crystallization was performed using the sitting-drop vapor diffusion method by mixing 0.5 µl of protein solution (5.0 mg ml^-1^ wild-type mIDH2 and 8.0 mg ml^−1^ K256Q mutant) and 0.5 µl of reservoir solution. Crystals of wild-type mIDH2 were obtained at 4 °C with a reservoir solution consisting of 0.1 M sodium citrate tribasic dehydrate (pH 5.6), 11.5% (v/v) 2-propanol and 10.8% (w/v) PEG 4000. Crystals of the K256Q mutant were obtained at 20 °C in a reservoir solution containing 0.1 M ammonium formate (pH 6.4) and 24% (w/v) PEG 3350.

Crystals were collected with a nylon loop and flash-cooled in a 95-K nitrogen stream. For cryoprotectants, crystals were soaked in a reservoir solution with 26% ethylene glycol for wild type and 22.4% ethylene glycol for the K256Q mutant. The X-ray diffraction data set was collected at the AR-NE3A beamline at the Photon Factory (Tsukuba, Japan).

### Structure determination and refinement

The diffraction data were indexed, integrated and scaled using the XDSAPP program^49^. The structure of mIDH2 was determined by molecular replacement with MOLREP^50^ using porcine IDH2 (PDB ID 1LWD; sequence identity of 96%) as a starting model. The obtained model was refined using Refmac5^51^ from the CCP4 program suite^52^ and ARP/wARP was used to build solvent^53^. ProSMART^54^ was utilized for the refinement of the structure of wild-type mIDH2, and twin refinement was used for the K256Q mutant. Iterative rounds of refinement were continued in Refmac5, making manual adjustments with COOT^55^. The visualization and superposition of protein structures were achieved using the PyMOL viewer (Version 1.5.0.4 Schrödinger, LLC). The surface electrostatic potential of the structures was calculated using the PDB2PQR server^56^ and PyMOL with the APBS plugin^57^. The CASTp server^38^ was used to calculate the volumes of protein cavities, using a probe radius of 1.4 Å. Data collection and refinement statistics are summarized in Table S1.

## Electronic supplementary material

Supplementary Information
