# Supplementary material for: Studies on the regulatory mechanism of isocitrate dehydrogenase 2 using acetylation mimics
Source: Sci Rep. 2017 Aug 29;7:9785. doi: 10.1038/s41598-017-10337-7 (PMC5575304; doi:10.1038/s41598-017-10337-7)
Supplement: Supplementary file 1 — Supplementary Information [file 41598_2017_10337_MOESM1_ESM.pdf]

Supplementary Information for

Studies on the regulatory mechanism of isocitrate dehydrogenase 2 using acetylation mimics

Yuqun Xu[1], Lingwen Liu[1], Akira Nakamura[1], Shinichi Someya[2], Takuya Miyakawa[1], Masaru Tanokura[1,*]

Affiliations:

[1]Department of Applied Biological Chemistry, Graduate School of Agricultural and Life Sciences, The University of Tokyo, 1-1-1 Yayoi, Bunkyo-ku, Tokyo 113-8657, Japan

[2]Departments of Aging and Geriatric Research, University of Florida, Gainesville, Florida, 32610-0143, United States of America

*Corresponding author. Email: amtanok@mail.ecc.u-tokyo.ac.jp

Contents

Supplementary Table 1: Statistics of X-ray collection data and refinement.

Supplementary Figure 1: Function of mIDH2 and mimic of acetylated mIDH2.

Supplementary Figure 2: Michaelis-Menten plot of the kinetics of wild-type mIDH2, the K256Q mutant and the K413Q mutant.

Supplementary Figure 3: Comparison of substrate binding mode of mIDH2 between subunit A and subunit B.

Supplementary Figure 4: Overlay of mIDH2 with porcine IDH2 and *E. coli* IDH in the vicinity of the putative phosphorylation.

Supplementary Figure 5: Isocitrate and Mg^{2+} binding residues between wild-type mIDH2 and the K256Q mutant.

Supplementary Figure 6: The putative $NADP^{+}$ binding pocket for mIDH2.

Supplementary Figure 7: Location of screened lysine residues in the structure of mIDH2.

Supplementary Figure 8: Crystal packing of wild-type mIDH2 and the K256Q mutant.

Supplementary Figure 9: The progress curve of reactions, related to Table 1 and Supplementary Fig. 2.

Supplementary Table 1. Statistics of X-ray data collection and refinement.

	**Wild-type mIDH2**	**K256Q mutant**
**Data collection**		
Beamline	PF AR-NE3A	PF AR-NE3A
Wavelength (Å)	1.0000	1.0000
Space group	$P3_221$	$P3_2$
Unit-cell parameters (Å)	135.9, 135.9, 220.2	58.2, 58.2, 206.9
Resolution range (Å)	45.9–3.29 (3.49–3.29)	45.28–2.21 (2.34–2.21)
No. of unique reflections	35964 (5579)	37204 (4577)
Redundancy	10.4 (9.7)	4.7 (2.2)
Completeness (%)	99.3 (97.0)	94.5 (71.7)
R_{meas} (%)	16.8 (123.1)	7.8 (35.3)
$CC_{1/2}$ (%)	99.9 (86.8)	99.7 (74.3)
$<I/\sigma(I)>$	14.1 (2.1)	16.0 (3.0)
**Refinement**		
No. reflections	33999	36284
R_{factor}/R_{free} (%)	19.2/22.2	20.9/24.3
No. atoms		
Protein	6612	6509
Isocitrate	26	26
Mg^{2+}	2	2
Water	4	128
B-factors ($Å^2$)		
Protein	117	32
Isocitrate	140	33
Mg^{2+}	96	33
Water	74	41
R.m.s. deviations		
Bond lengths (Å)	0.007	0.006
Bond angles (º)	1.04	1.08

*Values in parentheses are for the highest-resolution shell.

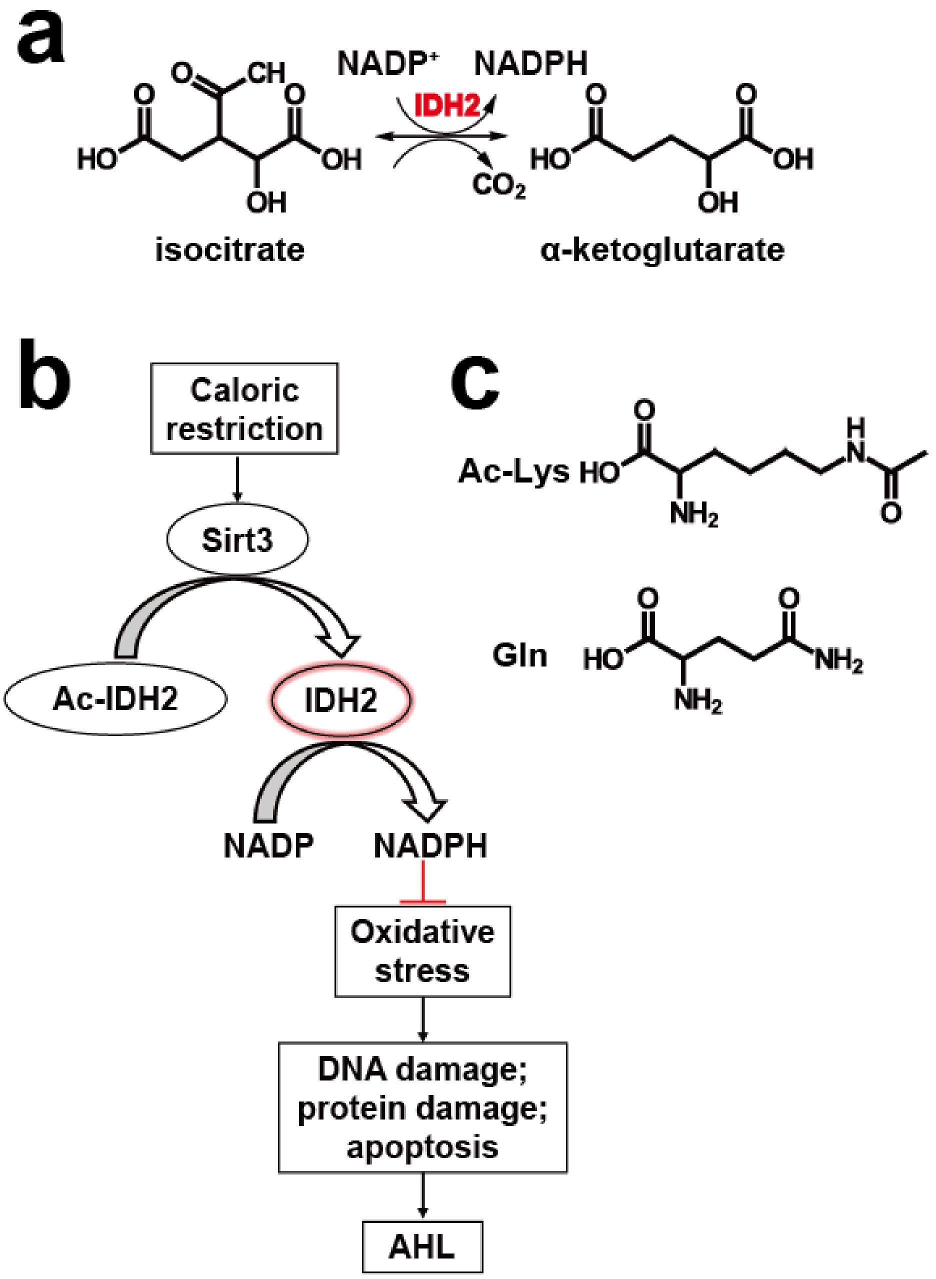

Supplementary Figure 1. Function of mIDH2 and mimic of acetylated mIDH2. (**a**) The reaction catalyzed by mIDH2. mIDH2 catalyzes the oxidative dehydrogenation and decarboxylation of isocitrate to α-ketoglutarate using $NADP^+$ as coenzyme, and Mg^{2+} as cofactor. (**b**) The model of the CR-mediated prevention of age-related hearing loss (AHL) in mammals[1]. (**c**) The similarity of acetylated lysine and glutamine.

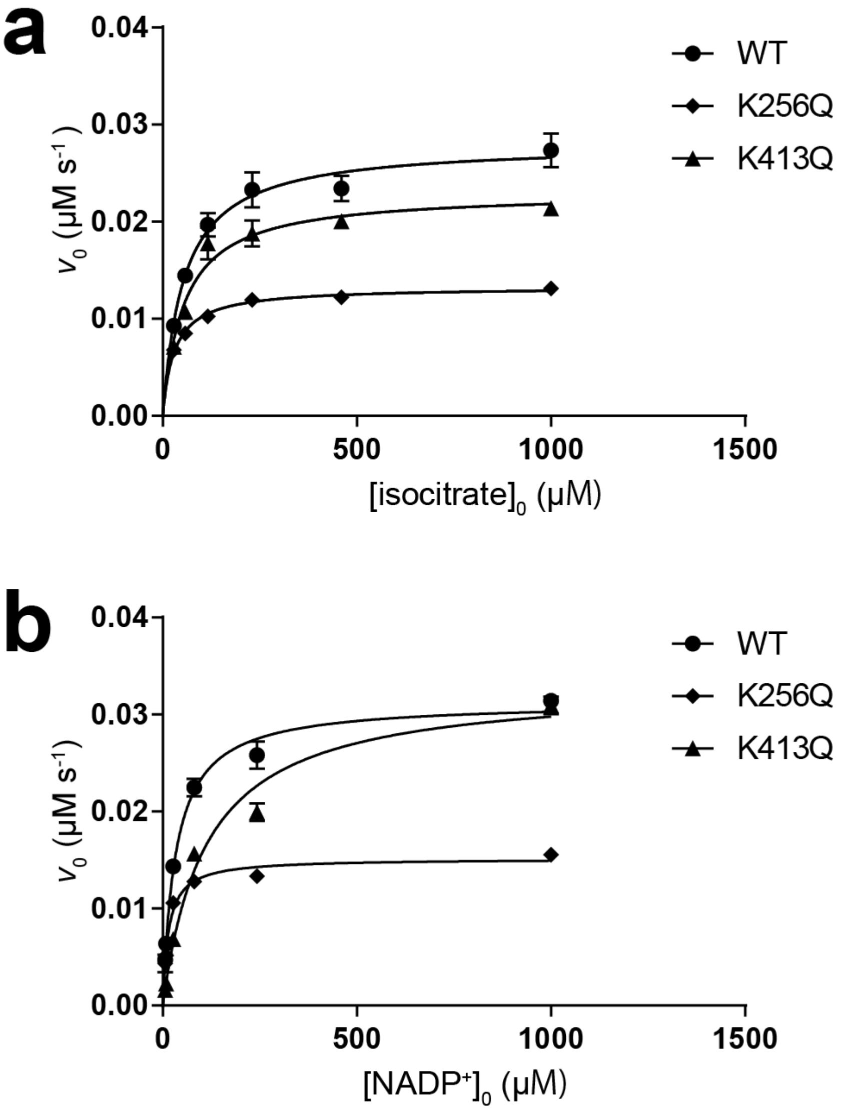

Supplementary Figure 2. Michaelis-Menten plots of the kinetics of wild-type mIDH2 (WT), and the K256Q and K413Q mutants. (**a, b**) The data of activity measured in several concentrations of isocitrate (**a**) or NADP$^+$ (**b**) are shown as the Michaelis-Menten plot (WT with round markers, the K256Q mutant with rhombus markers and the K413Q mutant with triangle markers). The data show means ± SD of four technically independent experiments.

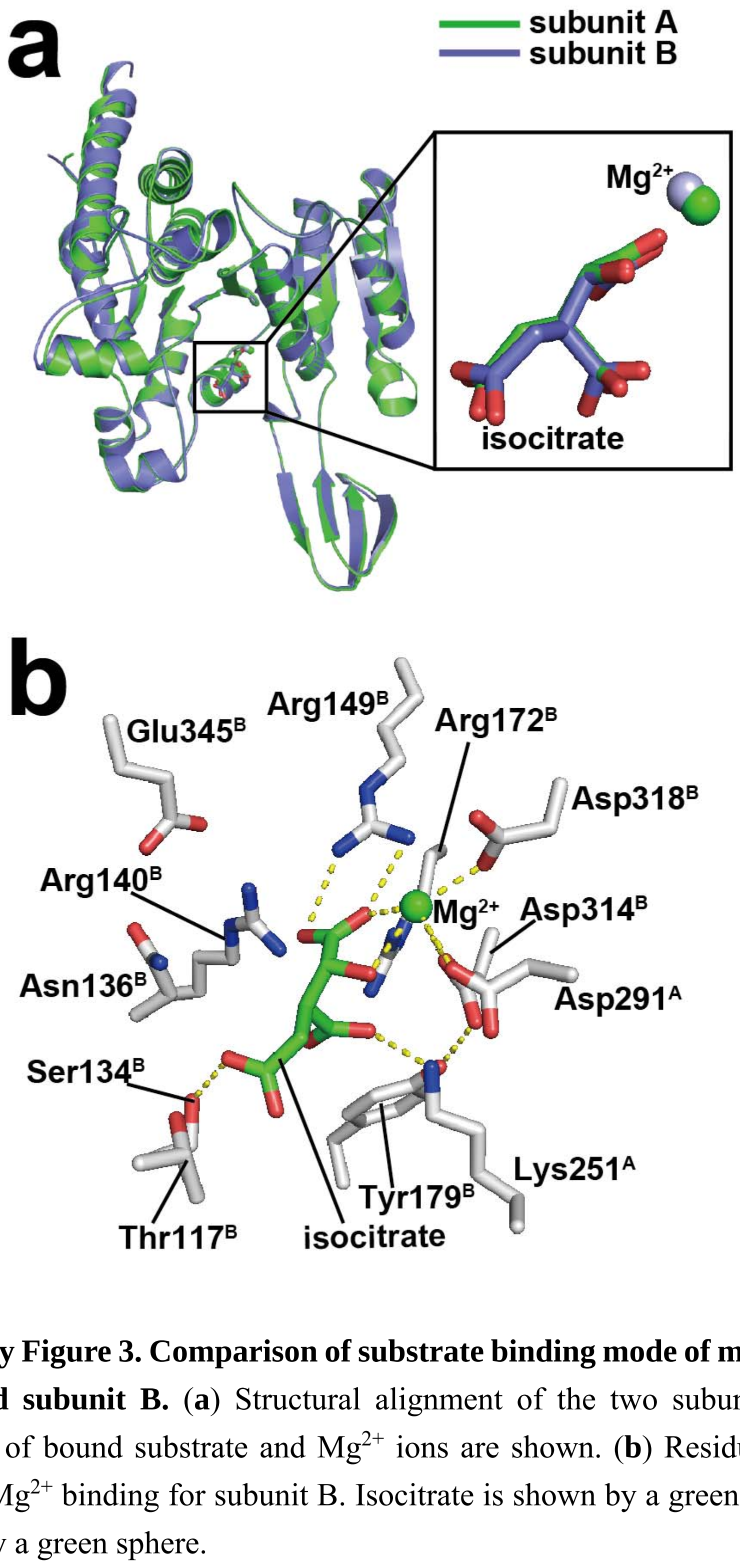

Supplementary Figure 3. Comparison of substrate binding mode of mIDH2 between subunit A and subunit B. (**a**) Structural alignment of the two subunits of mIDH2. Enlarged view of bound substrate and Mg^{2+} ions are shown. (**b**) Residues involved in isocitrate and Mg^{2+} binding for subunit B. Isocitrate is shown by a green stick and Mg^{2+} ion is shown by a green sphere.

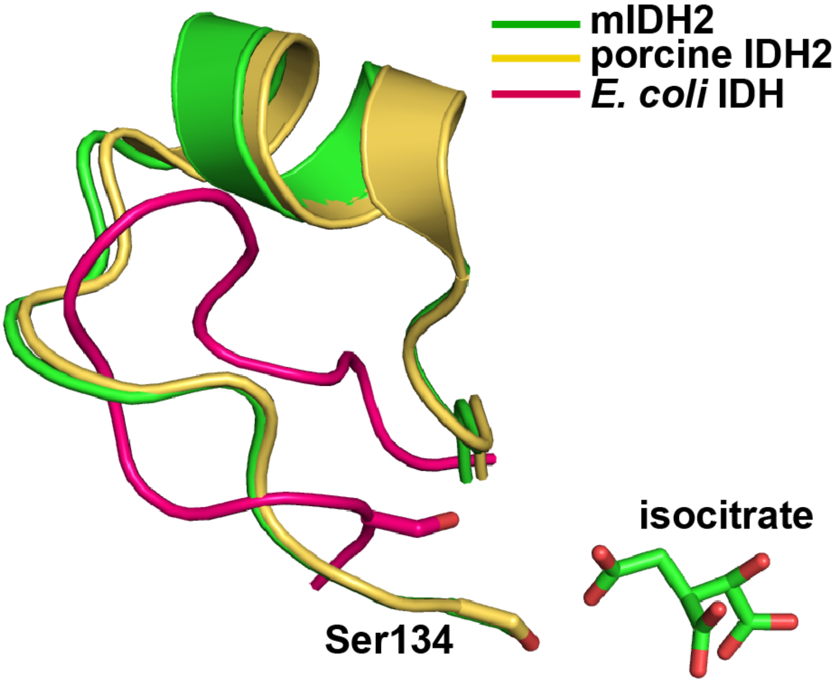

Supplementary Figure 4. Overlay of mIDH2 with porcine IDH2 and *E. coli* IDH in the vicinity of the putative phosphorylation site. The region of residues 115–134 is shown. This region of *E. coli* IDH (hot pink, PDB code: 1SJS) forms a loop while those of mIDH2 (green) and porcine IDH2 (yellow, PDB code: 1LWD) adopt α-helical conformation.

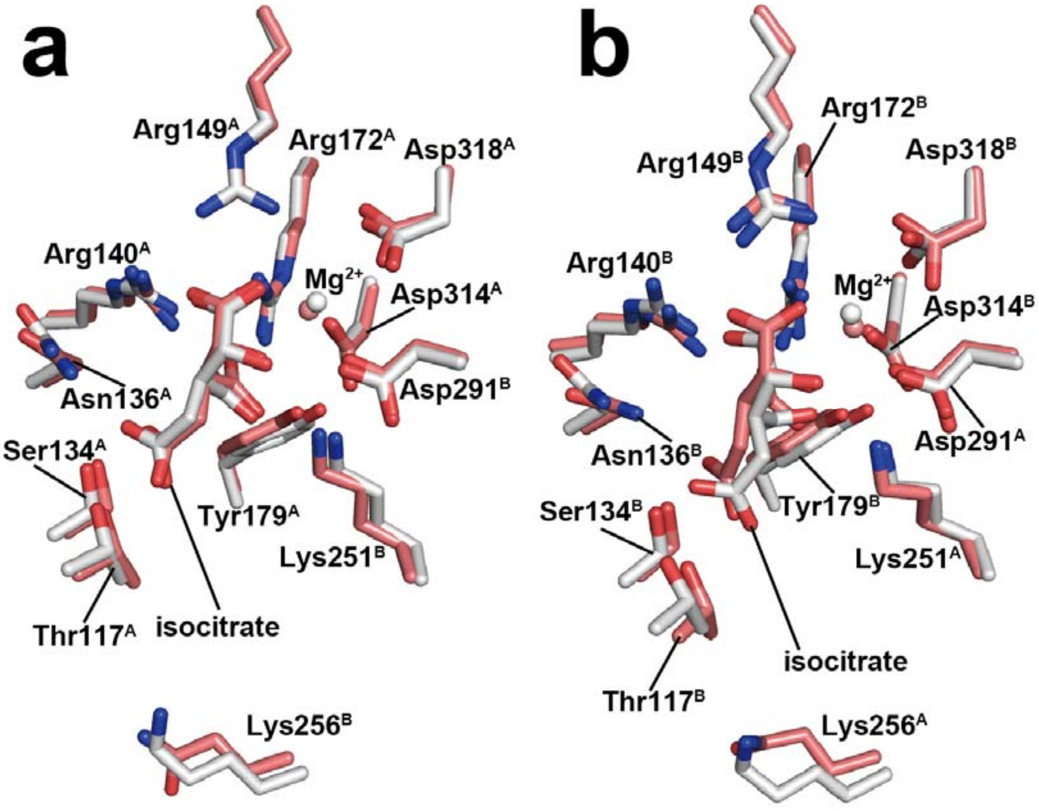

Supplementary Figure 5. Isocitrate and Mg^{2+} binding residues between wild-type mIDH2 and the K256Q mutant. (**a, b**) Detailed view of the isocitrate and Mg^{2+} binding residues (**a**, subunit A; **b**, subunit B) in the structural alignment. Amino acid residues, isocitrate and Mg^{2+} are shown in white for wild-type mIDH2 and pink for the K256Q mutant, respectively. Blue and red sticks represent nitrogen and oxygen atoms, respectively. Superscript capitals for amino residues represent subunit A or subunit B.

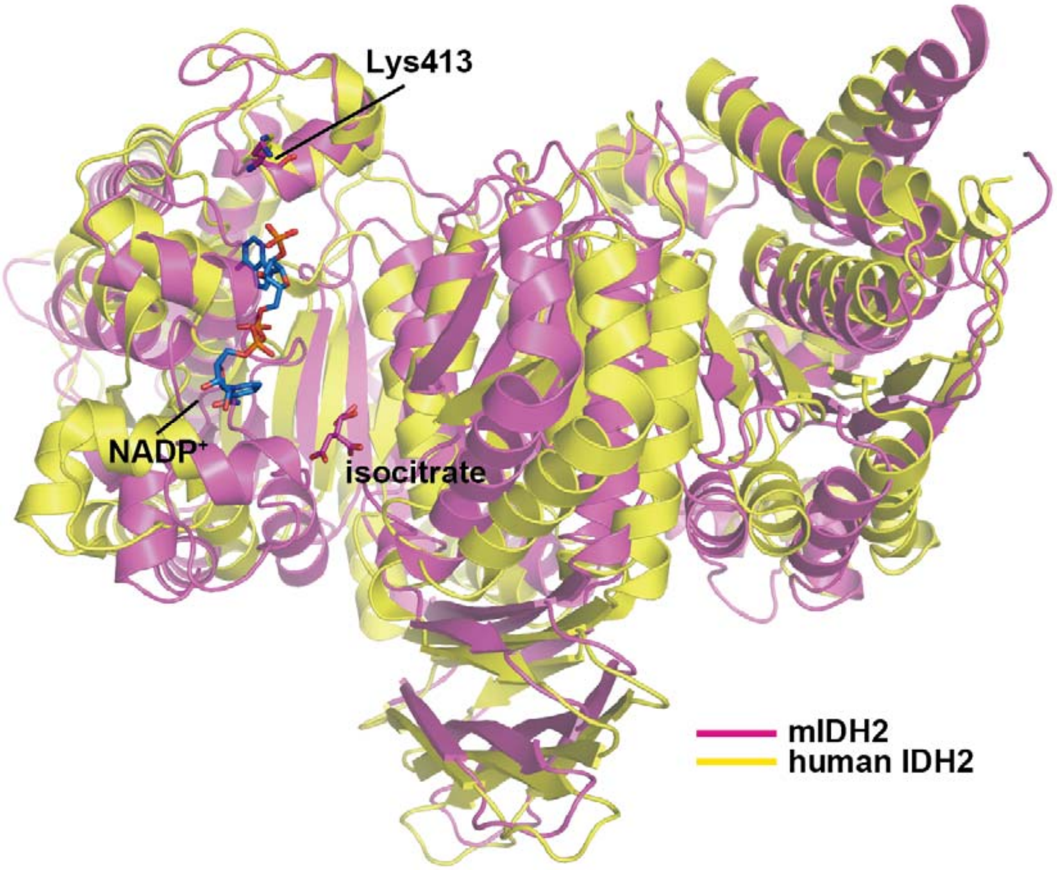

Supplementary Figure 6. The putative NADP$^+$ binding pocket for mIDH2. Overlay of mIDH2 and NADP$^+$-bound human IDH2 (open, PDB code: 4JAS) is shown. The structure of wild-type mIDH2 is shown in magenta and human IDH2 in yellow. NADP$^+$ from the structure of human IDH2 is shown by blue sticks. Lys413 is shown by stick form, suggesting that Lys413 is located near the NADP$^+$ binding site.

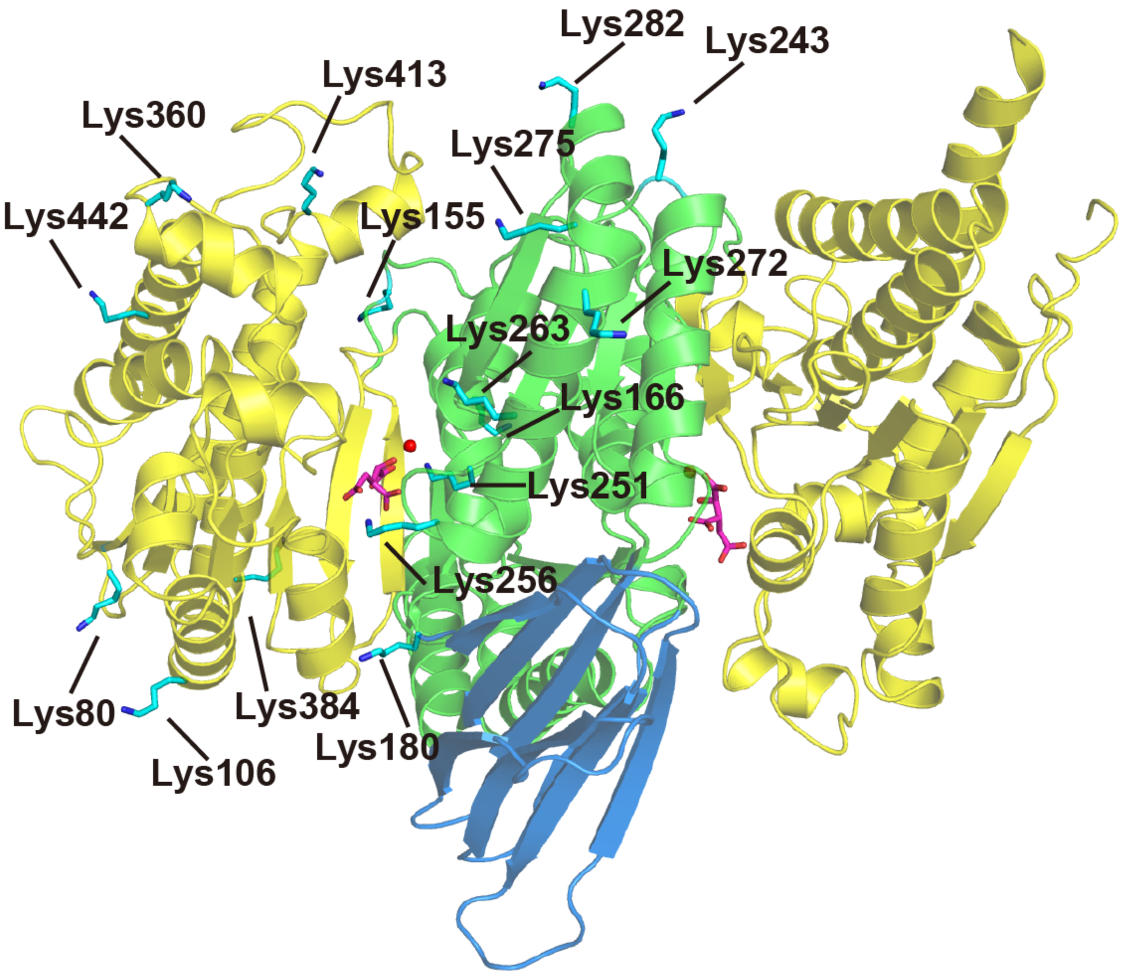

Supplementary Figure 7. Location of screened lysine residues in the structure of mIDH2. The large domain, the small domain and the clasp domain are colored in yellow, green and blue, respectively. Isocitrate and Mg^{2+} are shown in magenta. Lysine residues are shown by cyan sticks.

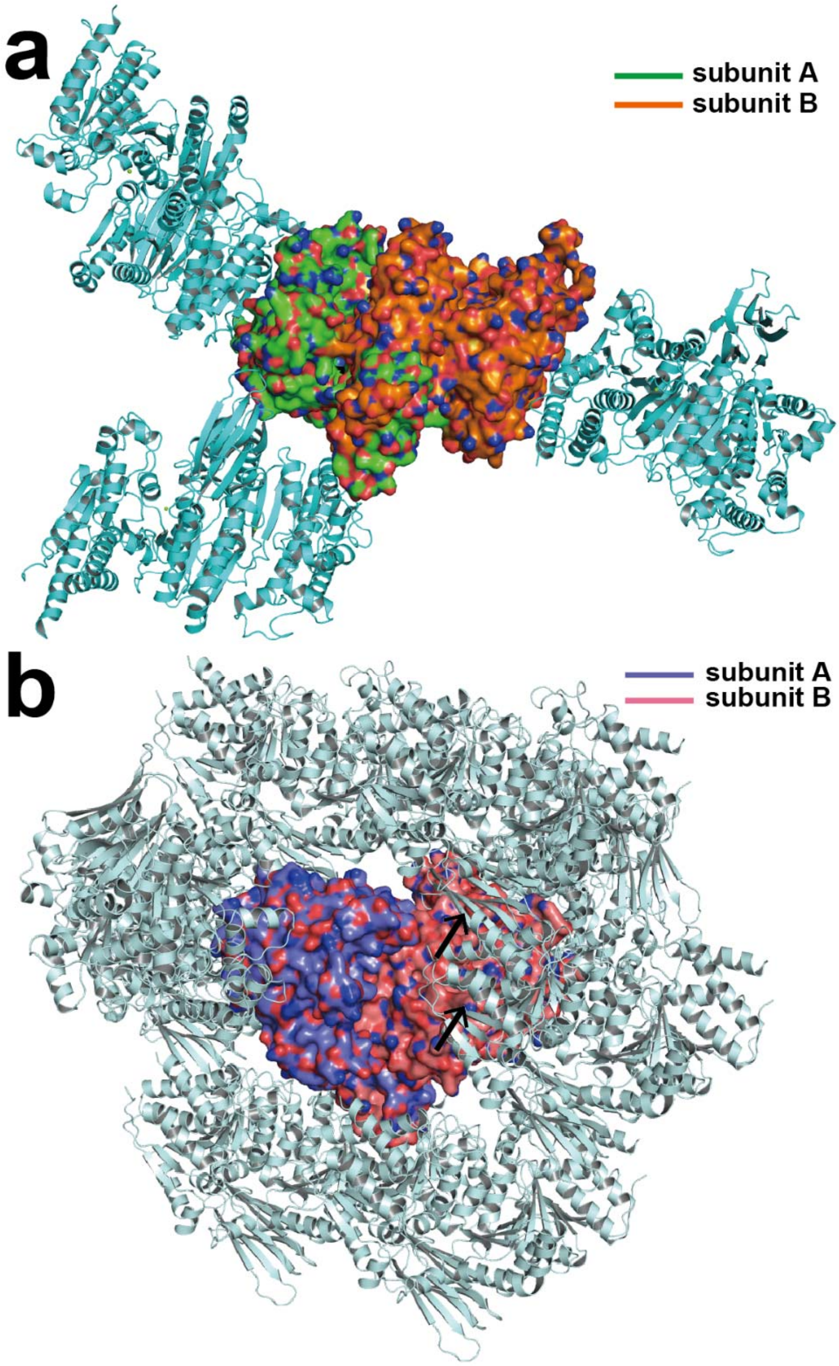

Supplementary Figure 8. Crystal packing of wild-type mIDH2 and the K256Q mutant. (**a**) Neighboring molecules within 4 Å in the crystal of wild-type mIDH2 have been shown in cyan. Subunit A is in green and subunit B in orange. (**b**) Neighboring molecules within 4 Å in the crystal of the K256Q mutant have been shown in pale cyan. Subunit A is drawn in slate blue and subunit B in pink. Black arrows highlight close crystal contacts between adjacent molecules.

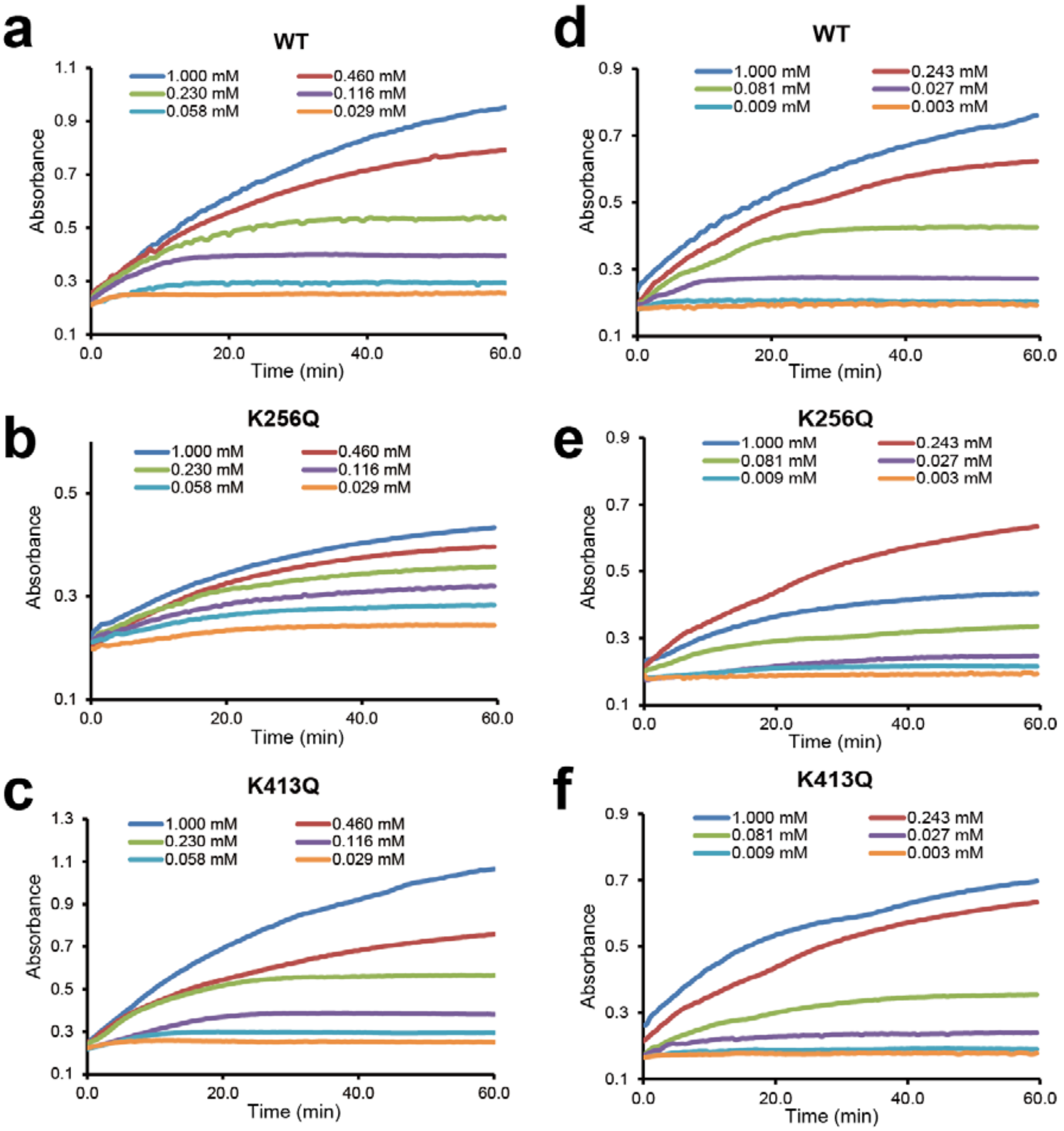

Supplementary Figure 9. The progress curve of reactions, related to Table 1 and Supplementary Fig. 2. (**a-c**) The curves of absorbance change measured in several concentrations of isocitrate with addition of wild-type mIDH2 (WT) (**a**), the K256Q mutant (**b**) and the K413Q mutant (**c**). Final concentrations of isocitrate have been indicated in the charts. (**d-f**) The curves of absorbance change measured in several concentrations of $NADP^+$ with addition of WT (**d**), the K256Q mutant (**e**) and the K413Q mutant (**f**). Final concentrations of $NADP^+$ have been indicated in the charts.

SUPPLEMENTARY REFERENCE

1. Someya, S. *et al.* Sirt3 mediates reduction of oxidative damage and prevention of age-related hearing loss under caloric restriction. *Cell* **143**,802–812 (2010).
